# A Comparison of Infant Feeding Practices in South Asian-Born Mothers and Australian-Born Mothers Living in Australia

**DOI:** 10.3390/nu16162577

**Published:** 2024-08-06

**Authors:** Durreajam Khokhar, Kristy Ann Bolton

**Affiliations:** Institute for Physical Activity and Nutrition, Deakin University, Geelong, VIC 3220, Australia; kristy.bolton@deakin.edu.au

**Keywords:** breastfeeding, formula feeding, complementary feeding, South Asian, immigrant, infant feeding, overweight, obesity

## Abstract

South Asian infants and children have a higher predisposition to central adiposity, increasing their risk of metabolic diseases in childhood. Infant feeding practices are a key factor in reducing the risk of obesity in children. The current study aimed to compare infant feeding practices of South Asian-born mothers to Australin-born mothers. The 2010 Australian National Infant Feeding Survey data were used to compare infant feeding practices between South Asian-born mothers and Australian-born mothers with children aged up to 2 years. Chi-square and t-tests were conducted, as well as regression models, with adjustment for covariates, to assess individual infant feeding practices between the two groups. A total of 298 South Asian-born mothers and 294 Australian-born mothers were included. The age at which a child stopped receiving breast milk was lower among Australian-born mothers (3 months) compared with South Asian-born mothers (5 months, *p* < 0.001). A greater proportion of South Asian-born mothers reported that solids were introduced at or after 6 months of age compared to Australian-born mothers (86% vs. 69%, *p* < 0.001, respectively). South Asian-born mothers were engaging in some health-promoting infant feeding practices compared to Australian-born mothers; however, they were not meeting the infant feeding guidelines for exclusive breastfeeding and the introduction of solids. Further research is needed to better understand factors influencing infant feeding practices in South Asian-born immigrant mothers in Australia to determine whether culturally tailored interventions are needed to help these women achieve optimal feeding practices for their infants.

## 1. Introduction

Overweight and obesity in children is a global public health issue, with 38 million children under 5 years of age being obese, indicating the high risk of this complex disease even among younger children [1]. In 2017–18, one in four Australian children and adolescents were considered to be living with overweight and obesity [2]. This is concerning as overweight and obesity in childhood is associated with an increased risk of developing chronic disease and mortality risk later in life [3,4,5]. It is noteworthy that the prevalence of obesity varies across ethnicities and may be linked with socioeconomic disadvantage [3,6,7,8]. 

Australia is known as the ‘immigrant nation’, with 30% of the population being born overseas [9]. South Asians are the fastest growing migrant population in Australia and represent 14% of the total overseas-born population [10]. The term ‘South Asian’ is an umbrella term and includes populations with origins from Bangladesh, India, Pakistan, Sri Lanka, Afghanistan, Nepal, Bhutan and the Maldives. Among the top five countries with the highest immigrant growth rates to Australia between 2006 and 2016, four were South Asian countries, including Nepal (27.8%), Pakistan (13.2%), India (10.7%) and Bangladesh (8.9%) [10,11]. Whilst this rise in immigration has increased Australia’s culturally and linguistically diverse population, it may also be contributing to the overall obesity rates, with evidence showing that children of immigrants have a greater risk of overweight and obesity compared to Caucasian children [6,7,12]. 

South Asian infants and children have a unique ‘thin fat’ phenotype, which is characterized by lower lean body mass and an increased body fat percentage despite being in a healthy weight range [7,13,14]. This phenotype is associated with excess central fat accumulation. In addition, South Asian children tend to have lower birth weights than Caucasian children but higher insulin resistance at birth and a rapid growth acceleration in early childhood [6,15,16]. These factors put South Asian children at an increased risk of obesity and other metabolic diseases in early childhood. 

Evidence suggests that the first 2000 days of life can have long-term consequences on health and that this is an important time to intervene and assist mothers in developing optimal infant feeding practices [17]. Breastfeeding is a significant protective factor in reducing childhood obesity [18]. Breastfeeding benefits both the infant and the mother [19]. In infants, breastfeeding provides adequate energy and nutrients as well as antibodies that reduce the risk of infection [19,20]. The long-term benefits of breastfeeding track into adulthood [21]. The current Australian Infant Feeding guidelines recommend exclusive breastfeeding for the first 6 months, when solids are introduced and continued until 12 months or beyond as desired by the mother and infant [22]. It has been shown that adults who were breastfed had lower rates of overweight and obesity, type 2 diabetes [21] and hypertension [23,24] than those fed infant formula. Evidence suggests that certain infant feeding practices may increase an infant’s risk of obesity, including overfeeding with infant formula, which can result in rapid weight gain leading to obesity later in life [25], and the timing of the introduction of solids, with the early introduction (<4 months old) of solids increasing the risk of overweight and obesity [26]. 

Infant feeding practices of Indian-born mothers living in Australia have been found to be suboptimal (e.g., infants are younger when exposed to sweetened water-based drinks and fruit juice) [27]. Furthermore, a systematic review of South Asian immigrants living in high-income countries also revealed early weaning and sweetened foods were being offered when initiating complementary feeding [28]. Infant feeding practices in immigrants can be influenced by a myriad of factors including, but not limited to, culture, traditions, beliefs, family support, language, education, healthcare professional support and socioeconomic status [29,30,31]. Acculturation may also influence infant feeding practices, such as the duration of breastfeeding and complementary feeding, in immigrant women living in Australia [32]. 

Given that South Asian children have a higher predisposition for central adiposity, which may increase the risk of metabolic diseases [6], the assessment of the feeding practices of South Asian-born mothers living in Australia is warranted. Therefore, the aim of this study was to compare infant feeding practices such as breastfeeding, infant formula feeding and the introduction of complementary feeding of South Asian-born mothers to Australian-born mothers living in Australia. 

## 2. Methods

### 2.1. Study Design and Participants

This was a secondary analysis of the Australian National Infant Feeding Survey (ANIFS), conducted by the Australian Institute of Health and Welfare (AIHW) [33]. The ANIFS, conducted during October 2010 and February 2011, provided cross-sectional baseline data on infant feeding practices, related to attitudes and behaviors of infants aged 0–24 months [33]. The infants were randomly selected from the national Medicare Australia enrolment database. Details pertaining to the methodology of this survey have been previously described elsewhere [33]. It should be noted that infants aged 6 months and above were oversampled to obtain quality data on breastfeeding duration for this age and for comparison with future survey data [33]. 

A total of 52,008 children aged up to 2 years (24 months) across Australia were selected randomly to be invited into the study. Children were included in the sample if they met the following criteria: at least one Medicare service or at least one record in the Australian Childhood Immunization Register in the past 12 months [33].

An initial letter detailing the objectives of the study was sent to the primary card holder on whose Medicare card the selected infant was listed [33]. One week later, a hard copy of the survey was sent by post, along with a reply-paid envelope. Participants were also given an option to complete the survey online. Two weeks following the initial approach letter, a reminder/thank you letter was sent to all survey participants (except those who opted out of the survey) [33]. A final letter was sent in the first week of December 2010 to remind any remaining non-responding participants to complete the survey [33]. 

**Figure 1:**
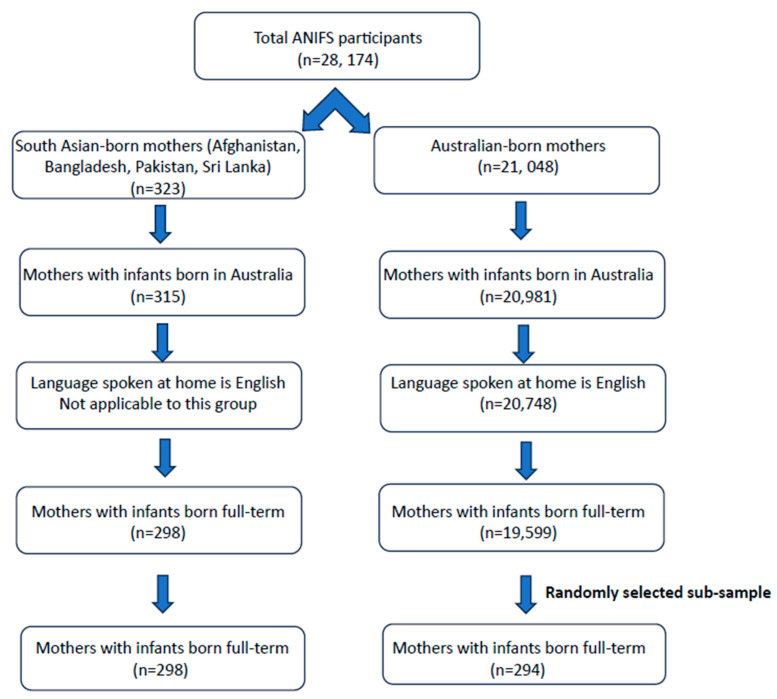
Flowchart of participant sampling from the Australian National Infant Feeding Survey.

The final sample was 28,759, giving a response rate of 56.4% (out of the 51,018 participants) [33]. Mothers were included in the analysis if they were born in either Afghanistan, Bangladesh, Pakistan, Sri Lanka or Australia. Mothers were excluded from the analysis if the infant was born overseas (*n* = 8 for South Asian-born mothers and *n* = 67 for Australian-born mothers), the Australian-born mothers did not speak English at home (*n* = 233) and if the infant was not born full term (premature: born < 37 weeks) (*n* = 17 for South Asian-born mothers and *n* = 1149 Australian-born mothers) (Figure 1). The final sample size of South Asian-born mothers meeting the inclusion criteria was *n* = 298. Given the large sample size of the Australian-born mothers (*n* = 19,599) a similar-sized sub-sample of mothers born in Australia was randomly selected to be included in this study by using a command in the statistical software program Stata version 15.0 (StataCorp LP, Lakeway, TX, USA). Therefore, *n* = 294 mothers born in Australia were randomly extracted from the total sample for analysis. 

### 2.2. Survey Instrument

The survey for the ANIFS was developed by the Australian Bureau of Statistics (ABS) to capture estimates on the prevalence and duration of breastfeeding, infant feeding practices and the barriers to initiating and continuing breastfeeding and perinatal depression as reported by mothers/carers of infants [33]. A review of the literature was conducted to inform the question design of the survey instrument and the survey was piloted tested, between August and September 2010, with parents of 1000 randomly selected children from the Medicare Australia enrolment database [33]. For further details of the piloting process of the survey, additional cognitive testing and the integrity of the survey instrument, please refer to the full 2010 Australian National Infant Feeding Survey Indicator Results report [33]. The final survey comprised 101 questions, 33 of which were utilized for the current study.

### 2.3. Sample Characteristics Surveyed 

The infant’s demographic information included the date of birth, country of birth, weight, length, type of birth (vaginal/cesarean section) and age in months. Maternal demographic information included the date of birth, country of birth, postcode, the main language spoken at home, relationship status, educational level, employment, total gross household income, smoking status (current), parity and weight and height after pregnancy. Socioeconomic status (SES) was based on the Socio-Economic Indexes for Areas (SEIFA) score of relative disadvantage [33]. This index includes quintiles based on income, education levels, employment status, marital status and competency in English, with the lowest quintile representing the most disadvantaged [33]. The ethnicity of mothers was determined by reported country of birth. For the purpose of this study, South Asian-born mothers were defined as being born in Afghanistan, Bangladesh, Pakistan or Sri Lanka and now living in Australia. 

### 2.4. Infant Feeding Practices Surveyed 

Mothers reported infant feeding practices and the ages (in months) when these practices were introduced. Mothers also reported current breastfeeding (yes/no) at the time of survey completion, whether the infant ever had breastmilk, formula, toddler milk, cow’s milk, soymilk, water, water-based drinks, fruit juice (yes/no) and the ages when these drinks were introduced. 

Water included any sips of water but excluded water combined with any other liquids such as cordial (a sweet non-alcoholic drink made from fruit juice) or solids (formula). Water-based drinks included cordial, soft drinks (non-alcoholic, carbonated/ non-carbonated, consisting of artificial colors, flavors and sugar) and tea. Cow’s milk included any sips of this milk, flavored milk and powdered milk but excluded these kinds of milk combined with solids (cereal). Soft, semi-solid and solid foods included custards, mashed foods and foods diluted with water, milk and other fluids [33]. 

### 2.5. Statistical Analysis

All data were analyzed using Stata/SE (StataCorp LP, Lakeway, TX, USA) version 15.0. Continuous and categorical data were expressed as the mean (±standard deviation (SD)) or number of participants and their percentage (*n*, %), respectively. Chi-square and *t*-tests were used to test categorical and continuous infant feeding practices between South Asian-born and Australian-born mothers. To examine the early introduction of water-based drinks, fruit juice and solids, age was dichotomized into <6 months (not meeting the recommendation) and ≥6 months (meeting the recommendation). To examine compliance with Australian infant feeding guidelines, age was dichotomized into <6 months (not meeting the recommendation) and ≥6 months (meeting the recommendation) for when breastfeeding was stopped and solids introduced, and <12 months (not meeting the recommendation) and ≥12 months (meeting the recommendation) for when cow’s milk was introduced. To assess for differences in infant feeding practices (dependent variable) by ethnicity (independent variable), infant feeding practices were broken down into dichotomized responses, and binary logistic regression was conducted with adjustment for covariates. Regression models were adjusted for maternal pre-pregnancy BMI, maternal SES (based on SEIFA) and total gross household income. For all analyses, a *p*-value of <0.05 was considered significant. 

## 3. Results

### 3.1. Demographic Characteristics 

A total of 298 South Asian-born mothers and 294 Australian-born mothers were included in the final sample (Table 1). Just over one third of South Asian-born mothers were aged between 30 and 34 years. The mean pre-pregnancy BMI was slightly lower in the South Asian-born mothers compared to the Australian-born mothers. A lower proportion of South Asian-born mothers were considered obese compared to Australian-born mothers. Over half of South Asian-born mothers had a tertiary education compared to just over one third of Australian-born mothers. One third of South Asian-born mothers were living in the most disadvantaged socioeconomic background compared with only twelve percent of Australian-born mothers. Thirty-six percent of South Asian-born mothers reported having a total gross income of $25,000 or below compared with only eight percent of Australian-born mothers. 

### 3.2. Infant Feeding Practices

The mean age at which the child stopped receiving any breast milk was lower among Australian-born mothers compared with South Asian-born mothers (Table 2). It is noteworthy that children born to South Asian-born mothers stopped receiving any breastmilk at around 5 months of age. South Asian-born mothers first provided the child with solid or semi-soft or solid foods at 5 months. Approximately three quarters of South Asian-born mothers reported that their child was currently receiving breastmilk compared with just over half of Australian-born mothers. Similarly, a higher proportion of South Asian-born mothers reported that their child was aged 6 months or older when they stopped receiving breast milk compared with Australian-born mothers. A little over one quarter of South Asian-born mothers reported introducing soft or semi-solid or solid food when the infant was less than 6 months of age. Comparatively, 43% of Australian mothers reported introducing soft or semi-solid or solid food when the infant was less than 6 months of age. 

A comparison of infant-feeding practices conducted by logistic regression showed they were mostly similar among the two groups of mothers, with the exception that infants of South Asian-born mothers had greater odds of stopping breastmilk at or after 6 months of age (odds ratio (OR) = 2.15, *p* = 0.003, adjusted for covariates) and that the child was currently receiving breastmilk at the time of the survey (OR = 1.85, *p* = 0.006, adjusted for covariates) (Table 3). Infants of South Asian-born mothers had lower odds of ever eating any soft or semi-soft or solid food (OR = 0.52, *p* = 0.007, adjusted for covariates).

## 4. Discussion

This study is the first study to assess infant feeding practices of South Asian-born mothers living in Australia and to compare these to Australian-born mothers. Overall, most of the infant feeding practices were similar for both groups, with the exception of the duration of breastfeeding, and the introduction of solids, for which South Asian-born mothers were more likely to be engaging in health-promoting infant feeding practices compared to Australian-born mothers. However, it should be noted that despite engaging in these more health-promoting practices, the majority of South Asian-born mothers, similar to Australian-born mothers overall, were not meeting the infant feeding recommendations and guidelines for the duration of breastfeeding, age of introduction to solids and the early age of introduction to water-based drinks and fruit juices. 

### 4.1. Breastfeeding

Whilst infants of South Asian-born mothers had higher odds of receiving breastmilk 6 months post-delivery and to be currently receiving breastmilk at the time of the survey compared to infants of Australian-born mothers, it should be noted that the mean age when breastfeeding was stopped was 5 months for infants of South Asian-born mothers. These infants fall short of meeting the recommendations of exclusive breastfeeding for the first 6 months of life and continued feeding until 12 months of age [22]. The introduction of infant formula at 1.5 months, on average, suggests the possibility of a combined feeding approach among infants of South Asian-born mothers. The literature assessing feeding practices among South Asian mothers living in high-income countries, particularly those born in Afghanistan, Bangladesh, Pakistan and Sri Lanka, is scarce but suggests that South Asian-born mothers living in the United States had higher breastfeeding initiation rates (89%) compared to African American women (51%) [34]. However, the rate declined to 39% at 6 months and 13% at 12 months [34]. Our findings showed that 85% of South Asian-born mothers continued breastfeeding beyond 6 months, although this study did not assess the prevalence of exclusive breastfeeding or whether breastfeeding was maintained up to 12 months or beyond. This may be a cause of concern because a prolonged duration of breastfeeding lowers the risk of obesity and cardiovascular disease-related conditions, such as hypertension, in children [35,36]. Breastfeeding duration is influenced by a myriad of factors such as maternal knowledge, attitudes, self-efficacy, as well as social and cultural factors [29]. Previous research in immigrant mothers shows that whilst they may be knowledgeable about the benefits of breastfeeding for the infant, they encounter several barriers related to successful exclusive breastfeeding such as breastfeeding in public spaces, conflicting advice from health professionals, changes to familial networks or the absence of family members and friends and access to infant formula [30]. Other factors such as language barriers, fear, depression and anxiety associated with migrating to a new country can also influence the duration of breastfeeding and introduction to infant formula [31]. Further exploration of the current breastfeeding practices, the rate of breastfeeding beyond 6 and 12 months and the role and impact of acculturation on feeding practices in South Asian-born mothers living in Australia is therefore warranted.

### 4.2. Infant Formula Feeding 

Our findings show that there was no difference, when adjusted for covariates, between the age at which infant formula was introduced and ever drinking any infant formula between infants of South Asian-born mothers and Australian-born mothers. Of note, three-quarters of infants of South Asian-born mothers had ever drunk any infant formula products and the mean age at which infant formula was introduced was 1.5 months. This study did not examine whether this was exclusive infant formula feeding or in combination with breastfeeding. The contradictory findings of breastfeeding beyond 6 months yet with the introduction of infant formula at just over one month of age may suggest a combination feeding approach among South Asian-born mothers. Overfeeding with infant formula may lead to rapid weight gain and a later risk of obesity and should be investigated in more detail exclusively [25]. In a qualitative study from the UK, South Asian mothers identified formula feeding as the most convenient method of feeding as this was a way to share the responsibility of feeding the infant (with other family members such as the husband or mother-in-law) and was less embarrassing than breastfeeding [37]. Formula feeding was opted for in response to misconceptions around fulfilling the infant’s nutritional needs or due to conflicting information provided by health professionals about the best form of feeding or conflicts with family members, particularly the mother and mother-in-law [37]. Further investigation into the barriers and facilitators of breast and formula feeding practices among South Asian-born mothers living in Australia are warranted. 

Whilst the Australian National Infant Feeding Survey did not collect data on the addition of sugary products to the bottle when feeding, evidence from a secondary analysis of dietary data from the Office of National Statistics survey on infant feeding in Asian families in the UK found that mothers were adding sugar and sugary foods to the bottle at 9 months, with 20% of Bangladeshi, 10% of Pakistani and 7% of Indian mothers engaging in this behavior [38]. The sugary foods added to the bottle included sugar, honey, rusks and chocolate powder. [38]. Given that South Asian children have a higher predisposition for central adiposity, which may increase the risk of metabolic diseases, future studies should assess the addition of sugary foods added to the bottle for infants of South Asian-born mothers living in Australia. The assessment of such behaviors may inform the development of culturally tailored educational resources and messages to support optimal feeding practices amongst South Asian immigrant mothers in Australia.

### 4.3. Introduction of Solids

Infants of South Asian-born mothers had lower odds of ever eating any semi-solid or solid foods compared to infants of Australian-born mothers. The average age of the introduction of solids did not differ between the two groups when adjusted for covariates (~5 months). Once again, infants of South Asian-born mothers were not meeting the infant feeding guidelines of introducing solids at 6 months of age. This could be a potential focus of future interventions as the early introduction of solids has been associated with an increased risk of overweight and obesity [26]. A study by Santorelli et al. found that Pakistani and other South Asian mothers living in Bradford, UK, were less likely to start complimentary feeding early (<17 weeks) compared to White British mothers [39]. However, South Asian mothers more frequently used sweetened foods as first complementary foods [39] compared to White British mothers. Similarly, in another study from Bradford, UK, Sahota et al. [40] reported that at 12 months of age, Pakistani infants consumed more commercially packed sweet baby food and potato chips than White British infants. Regarding the further investigation of the infant feeding practices of South Asian-born mothers living in Australia, it may be equally important to examine the timing as well as the *types* of solids introduced to ensure optimal feeding practices in this cohort. 

The early introduction of solids among infants of immigrant parents is influenced by family and cultural values [29,41]. Recent evidence from the UK suggests that Pakistani and Bangladeshi parents introduced solid food to infants between the age of 3 and 5 months, despite being advised by health professionals not to introduce complementary foods until 6 months of age [42]. The decision to introduce solids before 6 months of age was strongly influenced by cultural practices from the family’s country of origin. Another strongly influencing factor in initiating complementary foods was family (biological and in-laws) [42]. Amongst the Bengali and Pakistani families, the mother or mother-in-law would instruct the daughter or daughter-in-law about the timing of the introduction of solids based on their own experience [42]. Further research is needed to examine the role of familial and cultural practices in influencing the introduction of solids in order to provide culturally tailored advice to South Asian-born immigrant parents living in Australia. 

### 4.4. Water-Based Drinks and Fruit Juice

There was no difference in the timing of the introduction of water-based drinks and fruit juice, when adjusted for covariates, between infants of South Asian-born mothers compared to infants of Australian-born mothers. The average age for the introduction of water-based drinks was 6 months and 8 months for fruit juice among infants of South Asian-born mothers, suggesting yet another sub-optimal feeding practice. According to the Australian infant feeding guidelines, fruit juice is not recommended for infants under the age of 12 months as it displaces appetite for breastmilk and other solids and may increase the risk of damaging emerging teeth [22]. Furthermore, tea, herbal teas, coffee and other beverages do not offer any benefits to infants. In a study by Sahota et al. from the UK, Pakistani and other South Asian infants consumed more sugar-sweetened drinks and more fruit juice compared to White British infants [40]. Several studies have found that the consumption of fruit juice is associated with an increased risk of overweight and obesity in young children [43,44,45]. Recent evidence suggests that the consumption of sugar-sweetened beverages in infancy is associated with increased odds of obesity at the age of 6 [46]. 

### 4.5. Strengths and Limitations

This study was a secondary analysis of the Australian National Infant Feeding Survey, which included a large, randomly selected nationally representative sample of mothers, thereby increasing the generalizability of the findings to the wider Australian population. However, given that this survey was conducted thirteen years ago and the small sub-sample of the South Asian-born mothers, the findings should be interpreted with caution. Furthermore, the survey did not collect data specifically on exclusive breastfeeding and combination feeding (breastfeeding and formula feeding) and therefore may not be generalizable to the wider population. Due to the cross-sectional nature of the survey, causality cannot be inferred. However, these data form a baseline for comparison to future contemporary data on infant feeding behaviors in South Asian-born mothers living in Australia and are crucial for the development of intervention strategies to promote optimal infant feeding practices. Other limitations include the recall and social desirability biases which may be inherent in self-reported surveys and potentially unmeasured confounding factors (such as acculturation) and a lack of infant anthropometric data for the rate of growth. 

## 5. Conclusions

This study provides evidence of low compliance to health-promoting infant feeding practices in both Australian- and South Asian-born mothers living in Australia and a rationale for increasing resources and support for optimal infant feeding for all mothers. Given the growing South Asian immigrant population living in Australia, culturally appropriate early intervention is essential in helping South Asian-born mothers achieve optimal feeding practices for their infants and to reduce the future risk of overweight, obesity and chronic disease. 

## Figures and Tables

**Table 1:** Demographic characteristics of South Asian-born mothers and Australian-born mothers who participated in the Australian National Infant Feeding Survey (ANIFS).

	South Asian-Born Mothers (*n* = 298 *)	Australian-Born Mothers (*n* = 294 *)	*p*-Value
**Characteristics**	* n or mean (% or ±SD) *	* n or mean (% or ±SD) *	
Infant age at the time of survey completion (months)	6.4 (4.8)	7.0 (5.3)	0.17
Maternal pre-pregnancy BMI (kg/m^2^)	24.2 (4.5)	25.5 (5.9)	**0.009**
Maternal pre-pregnancy BMI category (kg/m^2^)			
Underweight (<18.5 kg/m^2^)	13 (5.9%)	12 (4.7%)	**0.007**
Healthy (18.5–24.9 kg/m^2^)	127 (57.7%)	132 (52.2%)	
Overweight (25.0–29.9 kg/m^2^)	60 (27.3%)	57 (22.5%)	
Obese (≥30 kg/m^2^)	20 (9.1%)	52 (20.6%)	
Maternal age group (years)			
15–24 years	17 (5.8%)	30 (10.2%)	0.18
25–29 years	93 (31.7%)	79 (26.9%)	
30–34 years	108 (36.9%)	112 (38.1%)	
35+ years	75 (25.6%)	73 (24.8%)	
Maternal highest level of education			
Tertiary education	171 (57.4%)	100 (34.0%)	**<0.001**
Diploma/certificate	56 (18.8%)	121 (41.2%)	
Year 12 or equivalent	31 (10.4%)	49 (16.7%)	
Year 11 or below	40 (13.2%)	24 (8.2%)	
Maternal Socioeconomic Status (SES)			
1st quintile (most disadvantaged)	96 (33.0%)	36 (12.3%)	**<0.001**
2nd quintile	39 (13.4%)	53 (18.2%)	
3rd quintile	58 (19.9%)	73 (25.0%)	
4th quintile	39 (13.4%)	60 (20.6%)	
5th quintile (most advantaged)	59 (20.3%)	70 (24.0%)	
Total gross household income (in past 12 months)			
$156,000 or more	11 (4.0%)	32 (11.2%)	**<0.001**
$88,400–$155,999	33 (12.1%)	101 (35.3%)	
$52,000–$88,399	56 (20.5%)	76 (26.6%)	
$26,000–$51,999	74 (27.1%)	53 (18.5%)	
$25,000 or below	99 (36.3%)	24 (8.4%)	
Maternal smoking status (current)			
Smoker	1 (0.40%)	26 (9.1%)	
Non-smoker	266 (99.6%)	259 (90.9%)	**<0.001**
Parity			
1 child	116 (43.8%)	122 (42.7%)	0.95
2 children	96 (36.2%)	104 (36.4%)	
3 or more	53 (20.0%)	60 (21.1)	
Spouse, husband or de facto partner living in the house			
Yes	277 (93.9%)	275 (94.2%)	0.89
No	18 (6.1%)	17 (5.8%)	
English is the main language spoken at home			
Yes	44 (15.0%)	294 (100%)	**<0.001**
No	250 (85%)	0 (0%)	

* N may not be 298 for South Asian-born mothers and 294 for Australian-born mothers as not all participants answered all questions. The ANIFS did not mandate responses for all questions.

**Table 2:** Infant feeding practices of South Asian-born women and Australian-born women who participated in the Australian National Infant Feeding Survey (ANIFS).

	South Asian-Born Mothers (*n* = 298 *)	Australian-Born Mothers (*n* = 294 *)	*p*-Value
**Infant feeding practices**	* mean (±SD) *	* mean (±SD) *	
Age when child stopped receiving any breastmilk (months)	5.3 (5.3)	3.0 (3.4)	**<0.001**
Age when child first drank infant formula products (months)	1.5 (2.3)	1.5 (2.2)	0.85
Age when child first drank cow’s milk (months)	10.5 (3.5)	10.8 (2.9)	0.66
Age when child first drank water (months)	3.4 (2.3)	3.5 (2.4)	0.74
Age when child first drank water-based ** drinks (months)	6.2 (4.8)	8.3 (5.8)	0.06
Age when child first drank fruit juice (months)	8.1 (4.6)	9.5 (4.9)	0.13
Age when child first ate soft or semi-soft or solid food (months)	5.1 (1.8)	4.7 (1.1)	**0.03**
	*N (%)*	*N (%)*	
Currently receiving breastmilk (incl. expressed milk or donor milk) (Yes)	214 (74.6%)	159 (56.8%)	**<0.001**
Age when child stopped receiving any breastmilk (months)			
<6 months	43 (14.4%)	91 (31.0%)	**<0.001**
≥6 months	255 (85.6%)	203 (69.0%)	
Ever drunk any cow’s milk (yes)	34 (14.4%)	49 (20.9%)	0.06
Ever drunk any infant formula product (yes)	171 (75.3%)	174 (77.7%)	0.56
Ever drunk any soy milk (yes)	4 (1.7%)	3 (1.3%)	0.71
Ever drunk any water-based drinks * (yes)	49 (20.8%)	40 (17.2%)	0.32
Age introduced water-based drinks * (months)			
<6 months	24 (8.1%)	14 (4.8%)	0.10
≥6 months	274 (91.9%)	280 (95.2%)	
Ever drunk any fruit juice (yes)	53 (22.6%)	55 (23.6%)	0.79
Age introduced fruit juice (months)			
<6 months	18 (6.0%)	10 (3.4%)	0.13
≥6 months	280 (94.0%)	284 (96.6%)	
Ever eaten any soft or semi-soft or solid food (yes)	130 (54.9%)	171 (73.7%)	**<0.001**
Age introduced soft or semi-soft or solid food			
<6 months	86 (28.9%)	127 (43.2%)	**<0.001**
≥6 months	212 (71.1%)	167 (56.8%)	

* N may not be 298 for South Asian-born mothers and 294 for Australian-born mothers as not all participants answered all questions. The ANIFS did not mandate responses for all questions. ** Water-based drinks: cordial, soft drinks, tea (excludes diluted fruit juice and infant formula products). Bolded values represent significance at *p* < 0.05.

**Table 3:** Comparison of infant feeding practices between South Asian-born women and Australian-born women who participated in the Australian National Infant Feeding Survey (ANIFS).

	South Asian-Born Mothers (*n* = 298 *)	Australian-Born Mothers (*n* = 294 *)	*p*-Value
**Infant feeding practices**	* OR (95% CI) *	* OR (95% CI) *	
Age when child stopped receiving any breastmilk (≥6 months)	2.15 (1.31–3.52)	1.00 (referent)	**0.003**
Age when child first drank infant formula products (≥6 months)	0.84 (0.56–1.26)	1.00 (referent)	0.40
Age when child first drank cow’s milk (≥12 months)	1.40 (0.56–3.56)	1.00 (referent)	0.47
Age when child first drank water (≥6 months)	1.21 (0.80–1.88)	1.00 (referent)	0.37
Age when child first drank water-based ** drinks (≥6 months)	0.67 (0.28–1.61)	1.00 (referent)	0.37
Age when child first drank fruit juice (≥6 months)	0.53 (0.21–1.30)	1.00 (referent)	0.16
Age when child first ate soft or semi-soft or solid food (≥6 months)	1.40 (0.93–2.11)	1.00 (referent)	0.11
Currently receiving breastmilk (incl. expressed milk or donor milk) (Yes)	1.85 (1.19–2.87)	1.00 (referent)	**0.006**
Ever drunk any cow’s milk (yes)	0.74 (0.42–1.32)	1.00 (referent)	0.31
Ever drunk any infant formula product (yes)	1.31 (0.75–2.27)	1.00 (referent)	0.34
Ever drunk any soy milk (yes)	1.33 (0.26–6.77)	1.00 (referent)	0.73
Ever drunk water (yes)	0.64 (0.40–1.05)	1.00 (referent)	0.07
Ever drunk any water-based drinks * (yes)	1.14 (0.64–2.04)	1.00 (referent)	0.66
Ever drunk any fruit juice (yes)	0.98 (0.59–1.64)	1.00 (referent)	0.94
Ever eaten any soft or semi-soft or solid food (yes)	0.52 (0.32–0.83)	1.00 (referent)	**0.007**

* N may not be 298 for South Asian-born mothers and 294 for Australian-born mothers as not all participants answered all questions. The ANIFS did not mandate responses for all questions. ** Water-based drinks: cordial, soft drinks, tea (excludes diluted fruit juice and infant formula products). Bolded values represent significance at *p* < 0.05. Logistic regression model adjusted for maternal pre-pregnancy BMI, maternal SES and total gross household income.

## Data Availability

The original data presented in the study from the 2010 Australian National Infant Feeding Survey is available for use with approval by the data custodian Australian Data Archive https://ada.edu.au/australian-national-infant-feeding-survey/.
